# Exploring the thermodynamics of disordered materials with quantum computing

**DOI:** 10.1126/sciadv.adt7156

**Published:** 2025-06-06

**Authors:** Bruno Camino, John Buckeridge, Nicholas Chancellor, C. Richard A. Catlow, Anna Maria Ferrari, Paul A. Warburton, Alexey A. Sokol, Scott M. Woodley

**Affiliations:** ^1^ Chemistry Department, University College London, 20 Gordon St, London WC1H 0AJ, UK.; ^2^ School of Engineering and Design, London South Bank University, 103 Borough Rd, London SE10 AA, UK.; ^3^ School of Computing, Newcastle University, 1 Science Square, Newcastle upon Tyne NE4 5TG, UK.; ^4^ Cardiff Catalysis Institute, School of Chemistry, Cardiff University, Park Place, Cardiff CF10 3AT, UK.; ^5^ UK Catalysis Hub, Research Complex at Harwell, Rutherford Appleton Laboratory, Harwell OX11 0QX, UK.; ^6^ Dipartimento di Chimica, Università degli Studi di Torino, Via Pietro Giuria 5, Torino 10125, Italy.; ^7^ London Centre for Nanotechnology, University College London, London WC1H 0AH, UK.; ^8^ Department of Electronic & Electrical Engineering, University College London, Torrington Pl, London WC1E 7JE, UK.

## Abstract

Alloys, solid solutions, and doped systems are essential in technologies such as energy generation and catalysis, but predicting their properties remains challenging because of compositional disorder. As the concentration of components changes in a binary solid solution $A_{\left(1-x\right)}B_{x}$ , the number of possible configurations becomes computationally intractable. Algorithms used in classical optimization methods cannot avoid assessing high-energy states where, for example, simulated annealing is designed to initially spend computational effort. We introduce a scalable, practical, and accurate approach using quantum annealing to efficiently sample low-energy configurations of disordered materials, avoiding the need for excessive high-energy calculations. Our method includes temperature and simulates large unit cells, producing a Boltzmann-like distribution to identify thermodynamically relevant structures. We demonstrate this by predicting bandgap bowing in ${\text{Al}}_{1-x}{\text{Ga}}_{x}N$ and bulk modulus variations in ${\text{Ta}}_{1-x}W_{x}$ , with results in excellent agreement with experiments.

## Teaser

A quantum annealer is employed to search for low energy states on complex energy landscapes of disordered materials.

## INTRODUCTION

A widely used strategy in materials design is to explore multicomponent disordered materials and to achieve optimal physical and chemical properties by modifying their composition. The compositional variation may result in amorphous structures, interstitial substitution, phase transitions, and the formation of defects. Often, crystallinity is maintained, and systems such as alloys, solid solutions, and heavily doped materials consist of crystalline materials in which one or more types of atoms, ions, or vacancies are incorporated into the crystal lattice of another substance without altering the overall crystal structure. These materials have many technological applications, including catalysis (*1*), energy storage (*2*–*4*), optoelectronics (*5*), nuclear fusion reactors (*6*), and lightweight materials (*7*).

The bulk phases of ordered crystalline phases can be uniquely defined by a unit cell with periodic boundary conditions, atom types, and their positions. Using this periodic model, a wide range of properties—including elastic constants, static and high-frequency dielectric constants, and phonon and electronic band structures—are routinely screened using computational chemistry and materials science techniques. Although the use of periodic models is ideal for ordered systems, it presents challenges in modeling disorder. In the study of disordered materials, we can consider random occupancy using analytical statistical approaches such as the virtual crystal approximation (VCA) (*8*, *9*) or coherent potential approximation (CPA) (*10*). Cluster expansion (CE) methods (*11*–*13*) include further refinement by explicitly modeling correlation effects. All these approaches are applicable to systems where species of the same oxidation state are mixed.

An alternative commonly used approach that accounts for long-range interactions, is not constrained to the same oxidation states, and is adopted in our work is to use a statistical ensemble approach and continue to use periodic boundary conditions applied to a unit cell to simulate an infinite number of atoms in the material. However, the limited number of atoms within the unit cell also restricts the total number of possible configurations that may be simulated and therefore may not capture the correct physics of the system. In such an approach, the unit cell should be increased to check that the physics is not dependent on the chosen simulation box size. Supercells are typically constructed from the primitive cell(s) of the end members and with the atom type of each site to be determined under the constraint of a chosen composition. The number of possible configurations depends on the ratio of the end members of the composition (e.g., a simple binary mix, $\chi_{x}^{A}\chi_{\left(1-x\right)}^{B}$ , has a maximum when *x* = 0.5) and grows factorially with respect to the number of sites in the cell and the number of different atom types. The chosen size of the supercell is restricted to what is manageable with the available computer resources, and the model works under the assumption that any key pattern can be represented or built from the patterns that are possible to model within the chosen supercell.

The main goal of the statistical analysis of disordered materials is to determine the configuration-energy relationship. In this approach, the effect of temperature can be explored as well as other external physical parameters. Thus, the aim is to consider as many of the thermodynamically accessible states as necessary to gain a sufficient representation of the system, or material of interest, as opposed to just targeting the ground state. The challenge here is to be able to generate and evaluate a manageable finite set of configurations that correctly represent the system, i.e., both qualitatively for the required physics and quantitatively for the required accuracy. With limited computer resources, there is tension between the level of theory used to evaluate each configuration and the number of configurations that can be evaluated. In practice, fewer states are sampled (because of the chosen size of the supercell), and a lower level of theory is used, for example, switching from an electronic structure approach to interatomic potential (IP) or machine-learned (ML) potential techniques. Energy functions that are cheaper to evaluate, such as model Hamiltonians, MLs, or IPs, can be parameterized or trained using a higher-level theory, which is pursued here. Choosing a smaller supercell may allow an exhaustive evaluation at a higher level of theory of the reduced sample space, particularly if symmetry is taken advantage of (*14*–*16*). When working with a larger supercell, for which an exhaustive search might not be possible due to the size of the configuration space, global optimization techniques such as Monte Carlo, simulated annealing, and genetic algorithms (*17*–*20*) can be used to identify low-energy configurations (which dominate the physics).

With the advance in quantum computation, a new family of optimization techniques are emerging where the search and evaluation of the potential energy landscape is conducted by a quantum computer. There are two relevant examples of combining approximated energy methods and classical, quantum-inspired, and quantum-optimization techniques that have proved successful in the configuration analysis of disordered materials. Choubisa *et al.* (*21*) used the CE method and a Fujitsu digital annealer (DA) to successfully perform the configuration analysis of quaternary Cu-Ni-Ag-Pd materials in a face-centered cubic (fcc) lattice. Furthermore, Gusev *et al.* (*22*) combined quantum annealing and continuous optimization to perform crystal structure prediction of ionic materials.

A key advantage of quantum annealing is that, under ideal conditions, it provides a guarantee of finding the global minimum of the objective function. Classical approaches—including Monte Carlo, simulated annealing, genetic algorithms, and machine learning–based methods—do not offer such guarantees and can become trapped in local minima, particularly in complex or high-dimensional energy landscapes. Although ML IPs and pattern-based optimizers can accelerate sampling, they typically require large training datasets and careful hyperparameter tuning.

In this work, we consider a system small enough (nitrogen-doped graphene) to demonstrate that quantum annealing can reliably recover low-energy configurations across a range of compositions. Although no quantum advantage is expected at this scale, the results serve as a proof of concept. As quantum hardware continues to improve in size, coherence, and connectivity, the ability to guarantee globally optimal solutions may become a significant advantage for materials discovery and design.

In general, at present, classical methods such as Monte Carlo, simulated annealing, and genetic algorithms outperform quantum approaches for configurational sampling, particularly in terms of scalability and solution quality. This is expected as both the algorithms and the supporting hardware for classical methods have been developed and optimized over several decades. By contrast, quantum annealing is a relatively recent technology. Nevertheless, when operated in the quantum coherent regime, quantum annealers (QAs) can outperform classical heuristic solvers (at least when quantified by certain performance metrics) in the simulation of the dynamical behavior of magnetic spin-glass materials at a scale of a few hundred spins (*23*). Although current quantum hardware does not yet offer a practical advantage for arbitrary very-large-scale materials problems, it is important to develop and test quantum-compatible algorithms now, in preparation for the point at which quantum devices reach the required level of maturity.

In the proof of concept presented in this paper, we show how to take advantage of D-Wave QAs (*24*) to explore the thermodynamics of disordered materials and crucially how we minimized the constraints to develop a model that works well on currently available annealers and scales much better than previously developed approaches, making complex systems accessible to our method. The technique we discuss provides notable improvements over recently developed classical and quantum methods for the study of disordered materials, enabling grand canonical simulations of large configurational spaces.

## RESULTS AND DISCUSSION

### Quantum annealing–assisted thermodynamic analysis of disordered materials

We present an approach that uses quantum annealing to explore the thermodynamics of disordered materials with a scalable model, advancing current methods. Quantum annealing is an optimization process designed to find the global minimum of a given objective function. It is particularly well suited to solving discrete combinatorial problems and has found applications across fields ranging from logistics to materials science. Quantum annealing belongs to the broader class of adiabatic quantum computing methods. The principle behind it is to encode the solution to a problem into the ground state of a quantum system, and then guide the system into this state by evolving its Hamiltonian slowly enough that it remains in or near the ground state throughout the process.

The physical implementation used in this work is based on D-Wave QAs (*24*), which use superconducting flux qubits arranged in a programmable network. Each qubit behaves like a quantum two-level system, realized using superconducting loops interrupted by Josephson junctions. The system is cooled below 20 mK to minimize thermal excitations and environmental noise. At the start of the annealing process, the qubits are placed in a transverse field that puts them into a quantum superposition of all possible states. As the transverse field is gradually reduced and the problem Hamiltonian is turned on, quantum fluctuations allow the system to tunnel through energy barriers, ideally ending in the ground state of the target Hamiltonian (where the problem of interest has been mapped to). In practice, imperfections and coupling to the environment may cause the system to evolve to a low-lying excited state. In this work, however, we show how the resulting Boltzmann-like distribution can be used to extract physically meaningful thermodynamic information.

A key constraint in superconducting flux-qubit quantum annealing is that only binary quadratic models can be directly mapped to the hardware. These include Ising spin models and Quadratic Unconstrained Binary Optimization (QUBO) problems, which are mathematically equivalent under a simple transformation. In the QUBO formalism, the system is described using binary variables $x_{i}\in \left\{0,1\right\}$ , and the objective function is expressed as

$$\begin{array}{c} E\left(x\right)=x^{T}\text{Qx}=\sum_{i}Q_{\mathrm{ii}}x_{i}+\sum_{i}\sum_{j>i}Q_{\mathrm{ij}}x_{i}x_{j}\;x_{i}\in \left\{\;0,1\;\right\} \end{array}$$ (1)

where the problem is encoded into the square matrix Q . The linear terms in the first summation originate from the binary nature of the $x_{i}$ variables, so $x_{i}^{2}=x_{i}$ because 0^2^ = 0 and 1^2^ = 1. In our work, the QUBO coefficients are derived from a physical model representing the chemical system of interest. The quadratic form captures pairwise interactions among sites in the supercell and can be viewed as a second-order expansion of the system energy around a binary configuration space. Although higher-order interactions exist in real materials, the QUBO model offers a tractable and hardware-compatible approximation that enables efficient sampling and optimization.

**Fig. 1.**
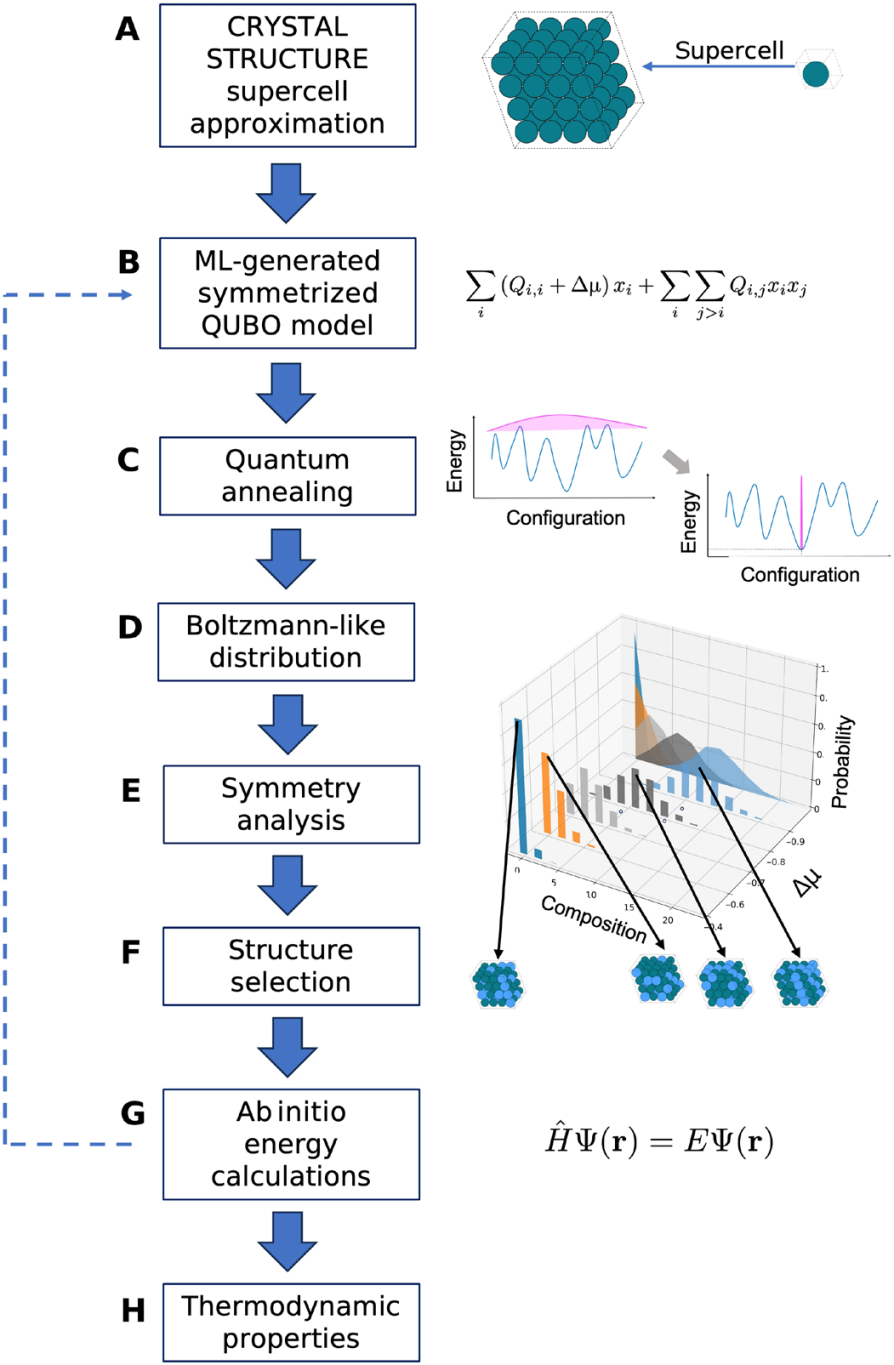
Workflow for the thermodynamic sampling of solid solutions using quantum annealing. Starting from the top: (**A**) Build a supercell from the unit cell of an end member. (**B**) Train the QUBO model using DFT data, for example, for a subset of configurations. A chemical potential Δμ , included in the QUBO model, is used to tune the composition. (**C**) Repeat use of D-Wave QAs to generate (**D**) Boltzmann-like probability distributions for structures within a range of compositions. (**E**) Perform the symmetry analysis of the structures returned by the QA to (**F**) find the unique configurations. (**G**) Check the quality of results by using a higher level of theory, such as DFT, on selected configurations from those identified (F) and, if necessary, return to step (B) using the structures (F) to train the QUBO model. (**H**) Use results from (G) to calculate thermodynamic properties.

The workflow used in this work, summarized in Fig. 1, starts from the definition of the crystal structure within the unit cell of an end member that is expanded to create a supercell that enables the exploration of mixed configurations. Each configuration is represented as a binary vector, and its energy is mapped to a QUBO model. The QUBO parameters are trained to reproduce results obtained from a small number of density functional theory (DFT) calculations on randomly generated configurations. By introducing a chemical potential, we can tune the composition of the resulting configurations to represent any stoichiometry of interest. As discussed below, by using the chemical potential rather than enforcing a fixed stoichiometry, our approach is better suited for currently available QAs, like those developed by D-Wave, which are characterized by limited qubit-qubit connectivity. A scaling factor in the QUBO model is used as a temperature parameter in our configurational analysis. In this setup, the QA works as a thermal sampler. By completing a series of quantum annealing runs, we obtain a Boltzmann-like distribution of QUBO energies. We demonstrate that these Boltzmann-like distributions closely represent those obtained from an exhaustive search. This is similar to previous quantum simulation works (*25*, *26*), but it extends these approaches to thermal sampling of solid solutions, an important topic in materials science.

To ensure that the analysis is carried forward on structures representing unique configurations, a symmetry analysis can be performed on the structures returned by the QA to obtain the subset of symmetry-independent configurations (SICs). The energy of the SICs can be determined at a more advanced level, such as DFT or through IPs. By repeating steps B to G in Fig. 1, we can train the model with low-energy structures instead of random ones, thus biasing it to explore low-energy regions of the configuration landscape. The structures obtained in step F can then be used to calculate the thermodynamic properties of the solid solutions in step H. For example, using the distribution of energies and structures taken from step F and the target property (structural parameters, elastic and dielectric response, phonons, electronic band structures, etc.) calculated as part of step G. An important advantage of the Boltzmann-like distribution returned by the QA is that it provides two complementary types of information. First, it identifies low-energy configurations that can serve as input for more accurate calculations. Second, it provides approximate Boltzmann weights that can be used to compute thermodynamic averages over configurations near a given composition, enabling direct comparison with experimentally measurable properties. The ability of the annealer to return accurate Boltzmann samples has been systematically quantified by Nelson *et al.* (*27*).

To show the versatility of the method, we apply our approach to three different types of materials, which have interesting technological applications: nitrogen-doped graphene (*28*, *29*) in catalysis and energy materials, ${\text{Al}}_{1-x}{\text{Ga}}_{x}N$ (*30*, *31*) in optoelectronics, and ${\text{Ta}}_{1-x}W_{x}$ alloys (*32*, *33*) used as structural components in nuclear and rocket systems due to their high temperature strength, high melting point, and good corrosion resistance. The supercells used to simulate these materials are depicted in the Materials and Methods section.

### Mapping the problem to a QUBO model

To solve a problem using quantum annealing, it must first be encoded into a form compatible with the constraints of the hardware. As discussed above, this means formulating the problem as a QUBO model, where the objective is to minimize a binary quadratic function.

In the context of configurational sampling in disordered materials, the unknown atomic pattern within the material of interest is defined on a lattice for which the site occupancies are to be determined. We map the supercell structure onto a binary vector x , where each element $x_{i}\in \left\{0,1\right\}$ represents the occupancy of site *i*. We focus on binary solid solutions, in which each site is occupied either by species $\chi^{A}$ or $\chi^{B}$ . In the case of ${\text{Al}}_{1-x}{\text{Ga}}_{x}N$ , for example, only the cation sublattice exhibits configurational disorder, and therefore only the cationic sites are treated as active degrees of freedom in the optimization.

In our mapping for the three selected systems, $\chi^{A}$ represents C in nitrogen-doped graphene, Al in ${\text{Al}}_{1-x}{\text{Ga}}_{x}N$ , and Ta in ${\text{Ta}}_{1-x}W_{x}$ , whereas $\chi^{B}$ represents N, Ga, and Ta, respectively. If *x_i_* = 0, $\chi^{A}$ is assigned to the lattice point *i*, and if *x_i_* = 1, it becomes $\chi^{B}$ . The QUBO model is constructed to yield an energy of 0 for pure graphene, AlN, and Ta (i.e., when all elements of the **x** vector are 0). Substituting $\chi^{A}$ atoms with $\chi^{B}$ atoms should result in an increase in energy. Therefore, a penalty is added for the presence of $\chi^{B}$ in $Q_{\mathrm{ii}}$ and another energy term, $Q_{\mathrm{ij}}$ , accounts for the interaction of $\chi^{B}$ with other neighboring sites, depending on which species occupies them. The task of the QA is to identify the binary vector x , obtained with such mapping, that minimizes the energy function, as defined in Eq. 1.

The QUBO model parameters $Q_{\mathrm{ii}}$ and $Q_{\mathrm{ij}}$ for the three targeted solid solutions are obtained using the linear regression of the DFT formation energies, of $N_{\text{train}}^{\text{SIC}}$ SICs with compositions in the range of stoichiometry of interest. The formation energy per active site (treated statistically) for the configuration *k* in the canonical ensemble is calculated as

$$\Delta {\tilde{E}}_{k}^{f}=\frac{1}{N_{\text{as}}}\left(E_{k}-\sum_{\chi^{i}}N_{k}^{\chi^{i}}E_{\chi^{i}}\right)$$ (2)

where $N_{\text{as}}$ is the number of active sites; $E_{k}$ is the total relaxed DFT energy per simulation cell of configuration *k*; $N_{k}^{\chi^{i}}$ is the number of sites occupied by species $\chi^{i}$ in configuration *k*; and $E_{\chi^{i}}$ is the energy of species *i* in its reference state. The reference states used in this work are pure graphene, molecular N_2_, AlN, GaN, Ta, and W. Each chosen configuration *k* is fully relaxed, that is, the training set only contains local minima. Further details of the DFT calculations and the optimization algorithm as implemented in the CRYSTAL23 code (*34*) and VASP (*35*) are provided in the Materials and Methods section. Although the QUBO model is defined on a set of lattice sites, the effects of the relaxation of atoms occupying these sites are implicitly included. Our QUBO model contains coupling constants, $Q_{\mathrm{ij}}$ , up to and including the next-nearest neighbors.

### Using the chemical potential to tune the composition

Consider using the basic QUBO model described above. As the QA is inherently an optimization machine, the configurations that are generated will typically be the trivial pure ground-state solution. The lowest QUBO energy structure, by construction, contains only $\chi^{A}$ atoms, that is, all the elements of the composition vector x are zero. However, here our interest is in mixed systems having a composition that is intermediate between the two end members. To model such compositions, one possible route is to impose a constraint on the number of $\chi^{B}$ atoms in the solution. Although this technique proved effective in crystal structure prediction (*22*), it results in a computationally expensive “all-to-all” fully connected QUBO model (as discussed in Materials and Methods). Because of the limited connectivity of current QAs, choosing fully connected QUBO models will result in long qubit chains and drastically limit the size of the problem that can be treated [see Appendix B of (*36*) for details]. By introducing the chemical potential, we can instead bias the solutions toward a specific concentration. The benefit of this technique is that it only adds diagonal elements to the QUBO model and therefore does not increase the connectivity (Materials and Methods).

In the chemical potential approach, we work within the grand canonical ensemble representation of the system. Consider a material in equilibrium with a reservoir containing its constituent species. Both the material and the reservoir are in equilibrium with a thermal bath. As the chemical potential of a particular species increases, there will be an increasing energy gain as an atom moves from the reservoir into the material. The relative energy of state *k* becomes

$$\Delta E_{k}^{f}=\Delta {\tilde{E}}_{k}^{f}+\frac{1}{N_{\text{as}}}\sum_{\chi^{i}}N_{k}^{\chi^{i}}\mu_{\chi^{i}}$$ (3)

where $\mu_{\chi^{i}}$ is the chemical potential of species $\chi^{i}$ and $\Delta {\tilde{E}}_{k}^{f}$ is taken from Eq. 2. If we consider only $N_{\text{as}}$ sites that are occupied by either $\chi^{A}$ or $\chi^{B}$ , then our energy term reduces to $\Delta {\tilde{E}}_{k}^{f\left(n\right)}+\mu_{\chi^{A}}+\frac{N_{\chi^{B}}}{N_{\text{as}}}\left(\mu_{\chi^{B}}-\mu_{\chi^{A}}\right)$ . The second term, $\mu_{\chi^{A}}$ , is constant for all configurations and can thus be ignored (or set to zero). The concentration of $\chi^{B}$ atoms predicted by the QA can therefore be controlled by adjusting the difference in the chemical potential, $\Delta \mu =\mu_{\chi^{B}}-\mu_{\chi^{A}}$ . The effect of Δμ on the QUBO energy levels is discussed in the Supplementary Materials and the “Selecting the desired composition” section of the Materials and Methods.

**Fig. 2.**
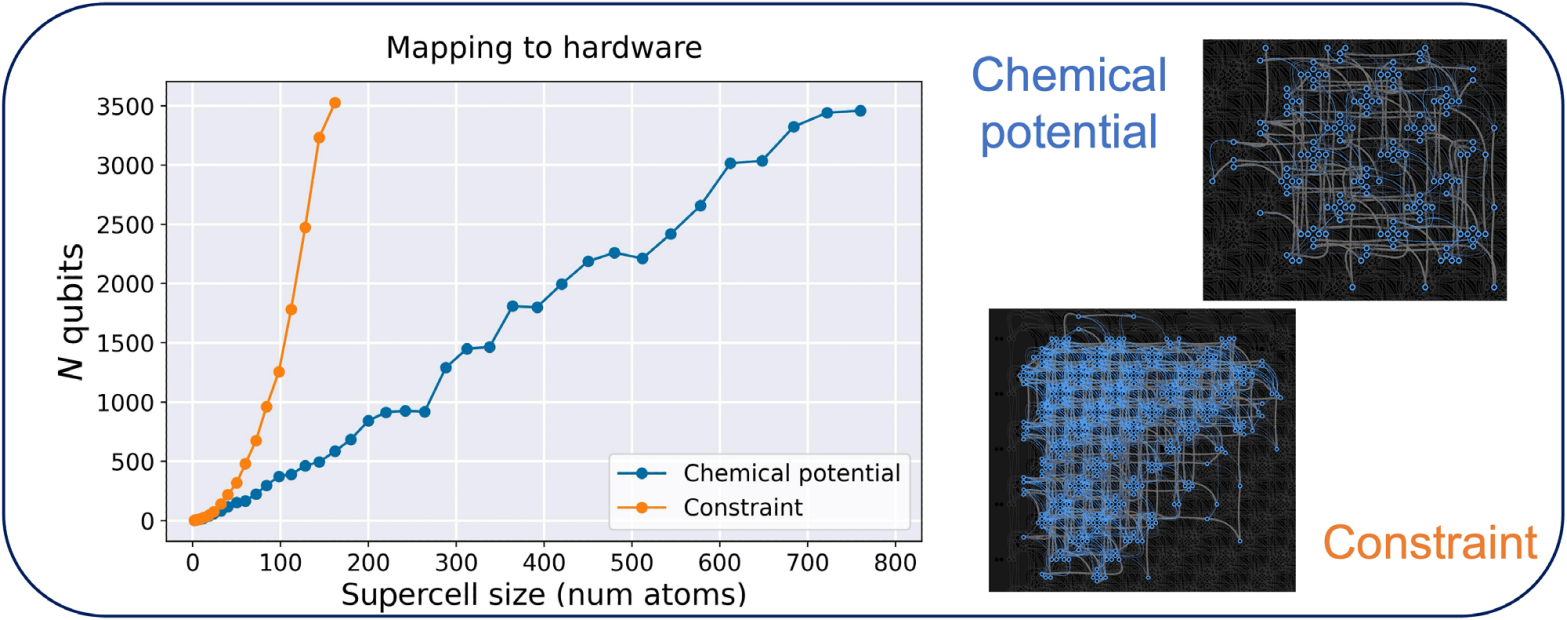
Mapping the problem to hardware. In the “mapping to hardware” panel, we plot the number of qubits required to map the QUBO problem for graphene to hardware when the chemical potential approach (blue line) and constraints (orange line) are used. The values were obtained by using the find_embedding function part of the minorminer library distributed via the D-Wave Ocean API. The images on the right show the physical qubits that the 50-atom graphene problem is assigned to on the QPU. These visuals were created with the dwave.inspector tool, which is included in the D-Wave API.

The number of qubits needed to implement both the fully connected and our QUBO models as a function of the problem size for graphene is displayed in Fig. 2. The optimal mapping is obtained heuristically using the minorminer library distributed as part of the D-Wave Ocean API (*37*). The blue data points show the number of qubits required to map the problem using the QUBO model defined in Eq. 4, which only has first and second nearest neighbor interactions (see Eq. 18). The orange data points show the number of qubits required to map the problem using a fully connected QUBO model to solve the same problem where the composition of the returned structures has been enforced by a penalty function [for more information on how to introduce the penalty function into the model, see ref. (*36*)]. When using the latter approach, the number of qubits quickly increases beyond the capacity of current QAs. The last orange data point corresponds to a 9 × 10 supercell containing 180 atoms. Mapping the next size (10 × 10 supercell) would require more qubits that are currently available on the Advantage QPU (quantum processing unit). On the other hand, using the former approach, we are able to map solid solutions based on a supercell containing up to 760 atoms. The images on the right side of the “mapping to hardware” panel show the physical qubits on the QPU needed to represent the 50-atom graphene problem by using the constraint and chemical potential approaches.

An aspect to consider in extending this approach to larger systems is the scaling behavior of the QUBO model and the limitations imposed by hardware connectivity. The current method scales linearly with the number of atomic sites in the supercell under the ideal condition where each site can be directly mapped to a single physical qubit. However, this scaling is not universal and depends on several factors. First, the effective connectivity of the model is that determined during its construction, particularly through the choice of how many neighbor shells are included in the QUBO model (as controlled by the parameter *n* in $A_{\mathrm{ij}}^{\mathrm{kn}}$ in Eq. 18). The number of interactions per site depends on the symmetry of the material and the local coordination environment. Systems with higher coordination numbers require more pairwise terms, increasing the qubit connectivity requirements. Second, scalability is constrained by the physical topology of the quantum annealing hardware. The present results were obtained using D-Wave’s Advantage topology, where each qubit is connected to up to 15 others. As future generations of QAs offer improved connectivity, the scaling of the chemical potential approach is expected to improve (i.e., the slope of the orange curve in Fig. 2 will become less steep).

An important advantage of the chemical potential approach over methods requiring all-to-all connectivity is that it offers flexibility: The model can be tuned to match the available hardware. For smaller systems or systems with simpler topologies, a greater number of neighbors (that is, *n* in $A_{\mathrm{ij}}^{\mathrm{kn}}$ in Eq. 18) can be used to capture more of the local environment. For larger supercells or systems with dense local bonding, *n* can be reduced to ensure that the QUBO model remains compatible with hardware constraints. In such cases, it is essential to validate the truncated model by assessing its accuracy on a test set, for instance, calculating the RMSE of the test set (as discussed in the Supplementary Materials).

To introduce the chemical potential into our QUBO model, we can fit the $Q_{\mathrm{ii}}$ and $Q_{\mathrm{ij}}$ terms using the canonical formation energies as defined in Eq. 2 and then add the Δμ term to the $Q_{\mathrm{ii}}$ terms so that the lattice sums in Eq. 1 become

$$E\left(x,\Delta \mu \right)=\sum_{i}\left(Q_{\mathrm{ii}}+\Delta \mu \right)x_{i}+\sum_{i}\sum_{j>i}Q_{\mathrm{ij}}x_{i}x_{j}\;x_{i}\in \left\{0,1\right\}$$ (4)

We treat the chemical potential as an adjustable parameter to tune the concentration of $\chi^{B}$ atoms in the structures returned by quantum annealing, but in a real experimental situation where a material is grown from constituent elements, constraints are placed on the allowed values of the chemical potential consistent with the formation of that material with respect to competing phases (*38*). In principle, we could relate Δμ to a realistic value with reference to our energy model of choice. However, doing so is not the focus of our current work as here we aim to demonstrate the applicability of the quantum annealing procedure to the analysis of disordered systems. A comprehensive analysis of the energetics of competing phases is beyond the scope of this study.

**Fig. 3.**
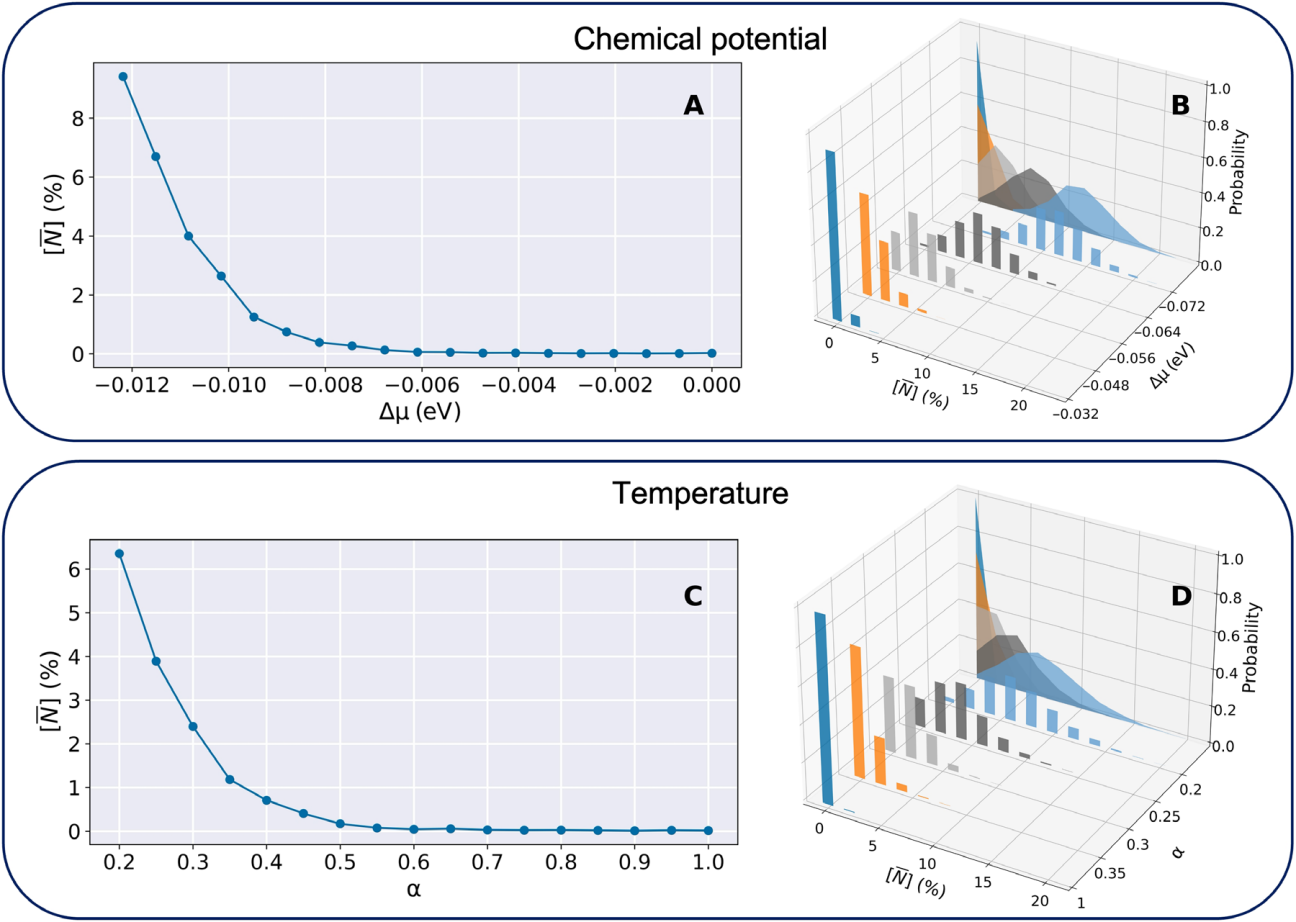
Analysis of the nitrogen concentration in a 50-atom graphene cell for a range of chemical potential and temperature values. The results displayed were obtained by postprocessing D-Wave quantum annealing data resulting from solving Eq. 4, where the QUBO model was trained from DFT data (Materials and Methods). The quantum annealing was run 1000 times. “Chemical potential” section: The plot in (**A**) displays the average nitrogen concentration as a function of the chemical potential. In (**B**), we plot the distribution of nitrogen concentration for five values of Δμ . The value of α = 1 was used to generate these plots. “Temperature” section: The plot in (**C**) displays the average nitrogen concentration as a function of the temperature introduced in the model through the α parameter. In (**D**), we plot the distribution of nitrogen concentration for five values of α. The value of Δμ = 0 was used to generate these plots.

We first illustrate the use of our chemical potential approach on nitrogen-doped graphene using D-Wave QAs. In Fig. 3A, we plot the average nitrogen concentration obtained for a range of Δμ values. For Δμ>−0.04 eV (noting that $Q_{\mathrm{ii}}$ = 0.08 eV), only pristine graphene structures are observed. For values of Δμ<−0.04 eV, structures containing nitrogen atoms are found. In Fig. 3B, we plot the nitrogen concentration distribution for five different values of Δμ . When Δμ=−0.032 eV, 90% of the structures found by the annealer are pristine graphene and 10% of the structures contain one nitrogen dopant. As the magnitude of Δμ increases, the likelihood of structures with a higher number of nitrogen atoms also increases, causing the peak of the distribution to shift toward higher concentrations.

### Introducing the temperature into the model

Temperature has been incorporated into our simulations. It is important to note that this temperature should not be mistaken for the hardware temperature, which, in the case of the D-Wave Advantage QPUs, remains below 20 mK. First, we consider what happens in the D-Wave experiments. If the annealer were operated at 0 K without nonadiabatic transitions, it would yield only the lowest energy state configuration. Operating at a finite temperature introduces the possibility of occupying excited states, resulting in a configuration that maximizes the system entropy, which means that the final-state distribution will follow a Boltzmann-like distribution (*39*–*42*).

One approach to thermally sample is simply to change the physical temperature of the annealer. This approach has been used in seminal D-Wave experiments (*43*), but it is neither practical nor available through the device API and, therefore, a different approach is required to incorporate temperature into our simulations. We achieve this by changing the energy scale of the problem by introducing a parameter that we define as α, which is inversely proportional to the temperature and has a range of values between 0 and 1 (*40*, *41*, *44*). Before running the quantum annealing, the D-Wave API scales the terms of the QUBO model to use the full energy range allowed by the annealer. From a Boltzmann distribution point of view, this corresponds to sampling at the lowest temperature permitted by the annealer. In this scenario, the global minimum (or minima) is more likely to be returned. However, in the context of solid solutions, we might be interested in a thermal sample at different temperatures, where the multiplicity of the states plays a role. To achieve this, the automated scaling option can be disabled and the $Q_{\mathrm{ii}}$ and $Q_{\mathrm{ij}}$ terms can be manually scaled using the parameter α.

The sample at the desired temperature can be obtained by trial and error by using several values of α until the desired temperature is obtained and the corresponding temperature obtained by postprocessing (*40*, *41*). Once two points are determined, any temperature between these points can be interpolated or points outside extrapolated.

Previous studies have demonstrated the utility of this approach for information theory tasks, which require a distribution that maximizes entropy under certain conditions (*39*) and for neural networks such as Boltzmann and Helmholtz machines (*45*–*51*). In this work, we focus on sampling distributions that span from low temperature regimes, where the ground state is more likely to be observed, to high temperatures, where state degeneracy becomes increasingly relevant.

We tested the effect of changing the values of α for the nitrogen-doped graphene system. In Fig. 3C, we show the average concentration of nitrogen atoms in the 50-atom graphene supercell as a function of α. For values of α > 0.6, most of the structures returned by the QA are pure graphene. This is the behavior that we expect at low temperatures, where only the ground state is observed. For values of α < 0.6, structures containing nitrogen dopants are found. In Fig. 3D, we plot the distribution of the nitrogen concentration of the structures for four values of α. As the temperature increases (α decreases), the distributions become flatter and shift toward a higher nitrogen concentration, as predicted by the Boltzmann distribution.

### Temperature and chemical potential regimes

In this section, we investigate the probability distributions generated by QAs as a function of both chemical potential and temperature. These are benchmarked against the complete set of configurations for the same supercell. Our goal is to establish the reliability of our quantum annealing approach, which can then be applied to larger and more complex systems. The chosen test system is nitrogen-doped graphene using the 50-site supercell depicted in the “Materials” section of the Materials and Methods. The exhaustive search, or complete set approach is manageable for investigating molar concentration of nitrogen atoms that is less than 15%. For 14% doping, there are more than 1.18 × 10^8^ configurations. Of these, there are a more manageable 397,227 SIC configurations, which we obtained using the configurational analysis tools implemented in the CRYSTAL23 (*34*) code.

**Fig. 4.**
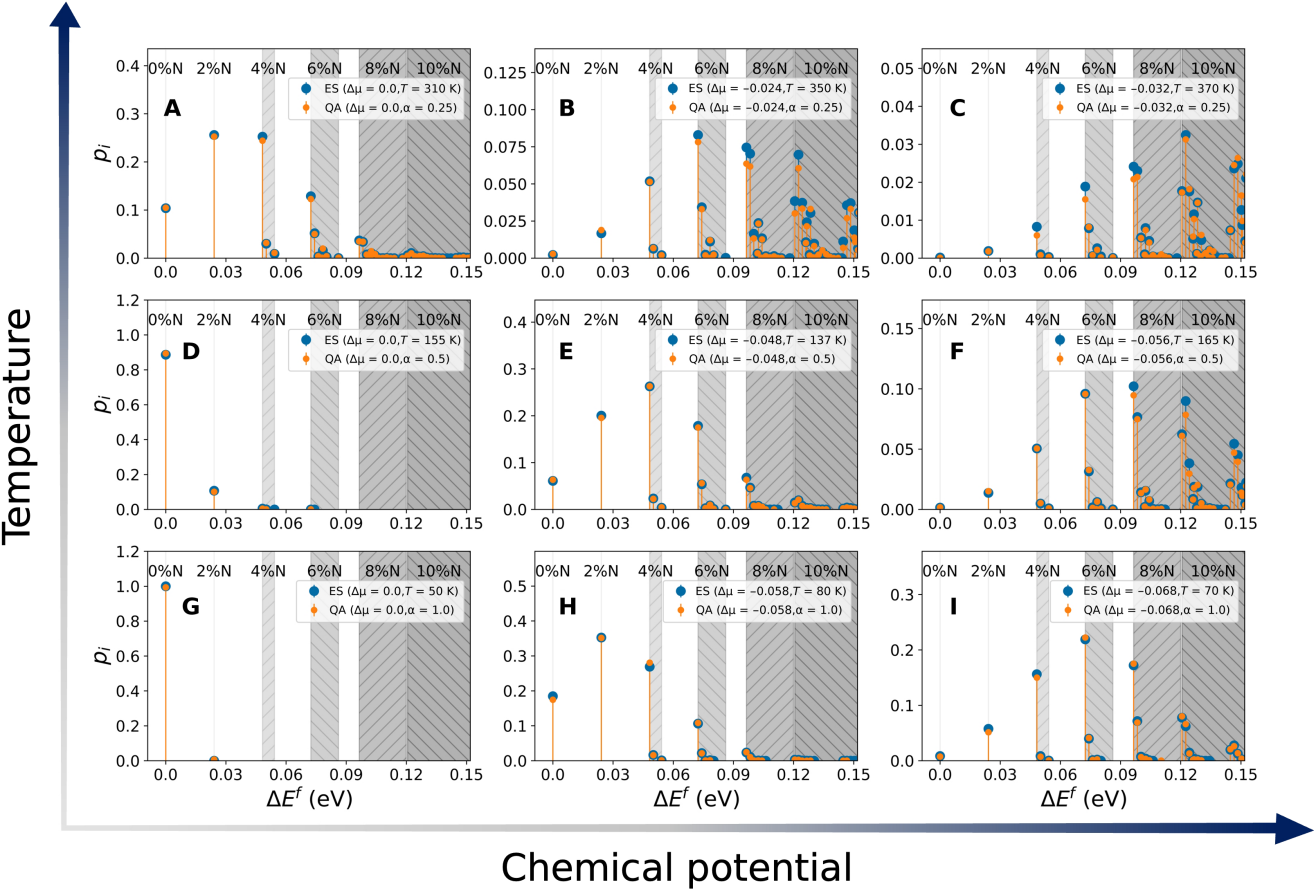
Probability distribution of nitrogen-doped graphene configurations within a 50-site supercell for representative values of chemical potential and temperature. (**A** to **I**) The orange and blue data points represent the data obtained from quantum annealing and from a complete set of possible configurations within the supercell, respectively. The gray-shaded areas indicate the nitrogen concentration in the configurations. Only configurations whose probability is higher than 1 × 10^−4^ are included. The temperature is reported in kelvin for the complete set, whereas α, which has a value between 0 (high temperature limit) and 1 (low temperature limit), is used in the quantum annealing runs. The Δμ value is given in units of eV.

In Fig. 4, we present the probability distributions collected using D-Wave QAs (depicted in orange) and those obtained classically from the exhaustive set of configurations (blue). To enable a visual comparison of probabilities across different sampling methods, such that the same structures always appear at the same energies in the graphs, the energies associated with each configuration *i* are plotted with respect to their relative formation energies $\Delta {\tilde{E}}_{i}^{f}$ , rather than the full grand canonical energies $\Delta E_{i}^{f}$ (these quantities are defined in Eqs. 2 and 3). This scaling effectively removes the contribution of the chemical potentials $\mu_{\chi^{i}}$ , ensuring that the ordering of the configurations is preserved. This is important because, although the Boltzmann probabilities are computed using $\Delta {\tilde{E}}_{i}^{f}$ , which includes $\mu_{\chi^{i}}$ , plotting the grand canonical energies would shift the positions of configurations depending on the choice of Δμ , making direct visual comparison across data obtained at different values of chemical potential difficult.

In the low chemical potential and low temperature regime, shown in Fig. 4G ( Δμ = 0.0, α = 1.0), the ground state configuration dominates, that is, only the pristine graphene structure is observed, as discussed earlier when considering Fig. 3. As the magnitude of Δμ increases, the likelihood of observing structures with higher concentrations of nitrogen also increases. For example, in Fig. 4I ( Δμ = −0.068 eV, α = 1.0), configurations containing 6% nitrogen dopants have the highest probability, followed by concentrations of 4 and 8%. Because these are in the low temperature regime, the lowest energy configuration for each of these compositions has the highest probability. This is an important aspect of these results because it shows that quantum annealing is able to find the ground-state energy configuration of nontrivial solid solution. For higher temperatures, the multiplicity of a state becomes a deciding factor on the probability of observing such a state, and even in the absence of chemical potential (Fig. 4, A and D), nitrogen-doped structures are possible. For high temperature and high chemical potentials, additional high-energy configurations are observed, which are not present in the low temperature and high chemical potential regime. For example, comparing Fig. 4 (C and I), within each concentration range, the probability of observing higher energy structures increases.

### Predicting the properties of real materials

In this section, we use the workflow outlined in Fig. 1 to predict the bandgap deviation from linearity for ${\text{Al}}_{1-x}{\text{Ga}}_{x}N$ , also known as the bandgap bowing and the bulk modulus for ${\text{Ta}}_{1-x}W_{x}$ structures. The QUBO model for both materials was generated using randomly generated structures across the entire range of composition (see Materials and Methods). These QUBO models produce a mean absolute error in the formation energy of 3.93 × 10^−4^ and 3.84 × 10^−6^ eV/unit formula for ${\text{Al}}_{1-x}{\text{Ga}}_{x}N$ and ${\text{Ta}}_{1-x}W_{x}$ , respectively.

**Fig. 5.**
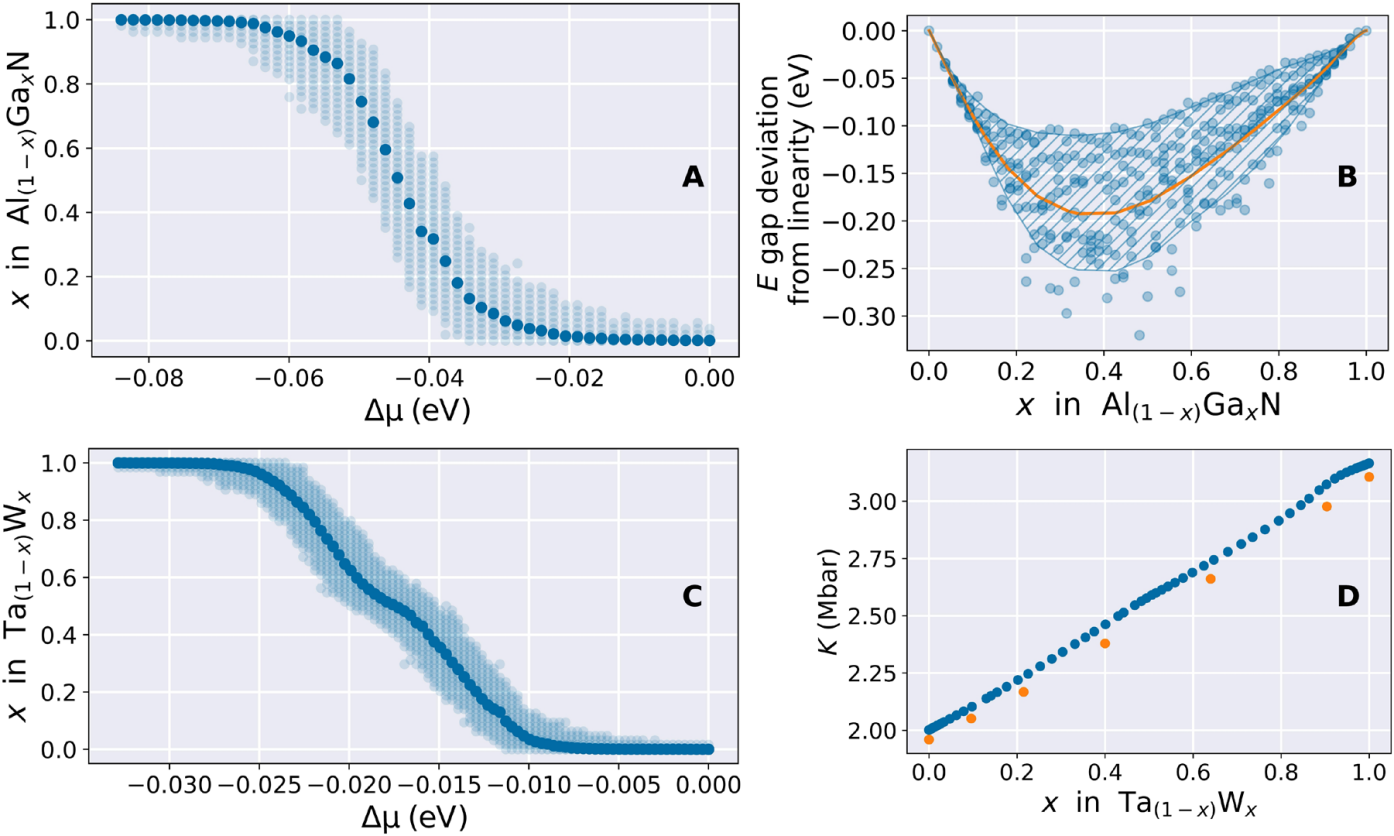
Quantum annealing–assisted prediction of real materials properties. (**A** and **C**) Average composition *x* in ${\text{Al}}_{1-x}{\text{Ga}}_{x}N$ and ${\text{Ta}}_{1-x}W_{x}$ as a function of the chemical potential Δμ (dark blue data points). The light blue points represent the interval of compositions returned by the QA. (**B**) ${\text{Al}}_{1-x}{\text{Ga}}_{x}N$ bandgap bowing as a function of the gallium content. The light blue points depict the values calculated for the low-energy configurations at each composition from ${\text{Al}}_{54}N_{54}$ cell to the ${\text{Ga}}_{54}N_{54}$ cell. The orange line depicts the bandgap bowing within the grand canonical ensemble calculated using Eq. 6. (**D**) Bulk modulus as a function of the tungsten ratio in ${\text{Ta}}_{1-x}W_{x}$ . The blue data points represent the values obtained within the grand canonical ensemble calculated using Eq. 7. The orange data points are the experimental values obtained from (*64*).

Quantum annealing was carried out over an interval of chemical potentials ranging from 0 to −0.12 eV for ${\text{Al}}_{1-x}{\text{Ga}}_{x}N$ and from 0 to −0.033 eV for ${\text{Ta}}_{1-x}W_{x}$ . The information we extract from the annealing is twofold. First, we select the low-energy structures obtained at the end of the annealing to perform more expensive ab initio calculations. Subsequently, we use the probability of observing specific configurations as an approximation of the Boltzmann weights to compute thermodynamic averages. To describe the materials more accurately, we simulate the property of interest for a range of configurations whose energy is below the thermal energy at room temperature (0.025 eV). We calculated the bandgap for 257 ${\text{Al}}_{1-x}{\text{Ga}}_{x}N$ structures using the CRYSTAL23 (*34*) code and the elastic tensor elements for 128 ${\text{Ta}}_{1-x}W_{x}$ structures using VASP (*35*). Therefore, we can calculate the average composition as a function of the chemical potential as follows

$$\langle x\rangle =\sum_{x=0}^{1}w_{x}x$$ (5)

where 〈x〉 is the average composition and $w_{x}$ is the fractional contribution of structures with composition *x*, both observed at potential Δμ . This grand canonical representation of the system allows for a more realistic description of the material in an experimental setting where the observed composition corresponds to the average of regions having varying compositions. The value of 〈x〉 relative to Δμ is represented by the dark blue data points in Fig. 5 (A and C). The lighter blue points represent the distribution of compositions obtained by the annealer around the average value.

We now focus on the calculation of the bandgap deviation from linearity for ${\text{Al}}_{1-x}{\text{Ga}}_{x}N$ , also known as the bandgap bowing, which breaks Vegard’s law. The light blue points in Fig. 5 represent the bandgap bowing calculated for the 257 structures selected from the low-energy configurations returned by the annealer. We then calculate the same property within the grand canonical ensemble representation of the system by using the same approach as in Eq. 5

$$E_{g}=\sum_{x=0}^{1}w_{x}E_{g}^{x}$$ (6)

where $E_{g}$ is the calculated bandgap, $w_{x}$ is the fractional contribution of structure ${\text{Al}}_{1-x}{\text{Ga}}_{x}N$ , and $E_{g}^{x}$ is the average bandgap for the lowest energy configurations having composition *x*, both observed at potential Δμ . The solid orange line in Fig. 5 depicts the average value of the bandgap bowing calculated using Eq. 6. The hatched area displays the bandgap $E_{g}^{x}$ region used in Eq. 6.

Assuming a parabolic dependence of the bandgap on composition, the bowing parameter *b* can be formulated using the equation: $E_{g}\left(x\right)=f\left(x\right)-\mathrm{bx}\left(1-x\right)$ , where $f\left(x\right)=\left(1-x\right)E_{g}\left(0\right)+{\mathrm{xE}}_{g}\left(1\right)$ is the linear dependence of the bandgap on composition. Here, $E_{g}\left(0\right)$ and ${\mathrm{xE}}_{g}\left(1\right)$ are the bandgap values of the end components. From the orange line in Fig. 5B, we calculate the bowing coefficient *b* = 0.67, which falls within the range of values *b* = 0.62 (± 0.45) (*52*) obtained experimentally.

The QUBO model constructed from DFT-derived formation energies has positive $Q_{\mathrm{ij}}$ interaction terms. This coupling energetically favors configurations in which neighboring sites are occupied by different atom types (i.e., Al-Ga pairs), thereby promoting local disorder in the resulting solid solutions. This preference for disorder is consistent with the well-established explanation for the bandgap bowing observed in ${\text{Al}}_{1-x}{\text{Ga}}_{x}N$ solid solutions. The difference in electronegativity and atomic potentials between Al and Ga atoms leads to fluctuations of the electronic potential landscape. These fluctuations introduce a nonlinear perturbation to the band structure, particularly at the band edges, which manifests itself as a downward bowing of the bandgap as a function of composition (*53*). Random alloys, which maximize such disorder, exhibit stronger bowing than ordered configurations (*54*).

The same approach was used to calculate the bulk modulus (*K*) of ${\text{Ta}}_{1-x}W_{x}$ , with the findings presented in Fig. 5D. The bulk modulus determined using the grand canonical approach is given by

$$K_{g}=\sum_{x=0}^{1}w_{x}K_{g}^{x}$$ (7)

where $K_{g}$ is the calculated bulk modulus, $w_{x}$ is the fractional contribution of structure ${\text{Ta}}_{1-x}W_{x}$ , and $K_{g}^{x}$ is the average bulk modulus for the lowest energy configurations having composition *x*. Despite a minor overestimation of the bulk modulus, also observed for pure Ta ( $\Delta K_{\text{exp}-\text{DFT}}=4.21\times 10^{-2}$ Mbar) and W ( $\Delta K_{\text{exp}-\text{DFT}}=5.99\times 10^{-2}$ Mbar), the trend of the bulk modulus with respect to the W ratio calculated using the grand canonical method aligns closely with the experimentally observed data, thus further validating this method.

The workflow we developed can be applied to the study of any material with substitutional disorder, regardless of the energy method used to build the QUBO model. It is tailored to current D-Wave QAs, which have limited connectivity, by creating a QUBO model that only includes interactions with the nearest and next-nearest neighbor sites in the lattice and introduces the chemical potential to tune the composition of the material. This model requires fewer qubits to run on QAs than a QUBO model with penalty terms, making it suitable for large supercells. Furthermore, we illustrated that this method offers significantly improved scaling compared to the commonly used constrained QUBO model approach. We demonstrated how the result of running the quantum annealing multiple times can be interpreted as a Boltzmann distribution within the grand canonical ensemble, and the QUBO terms can be scaled to explore the configurational space at different temperature regimes. The method we presented offers substantial advantages over previously developed classical and quantum approaches in the study of disordered materials allowing for the grand canonical simulations of configurational spaces whose size is too large to be tackled by classical computers.

## MATERIALS AND METHODS

### Materials

**Fig. 6.**
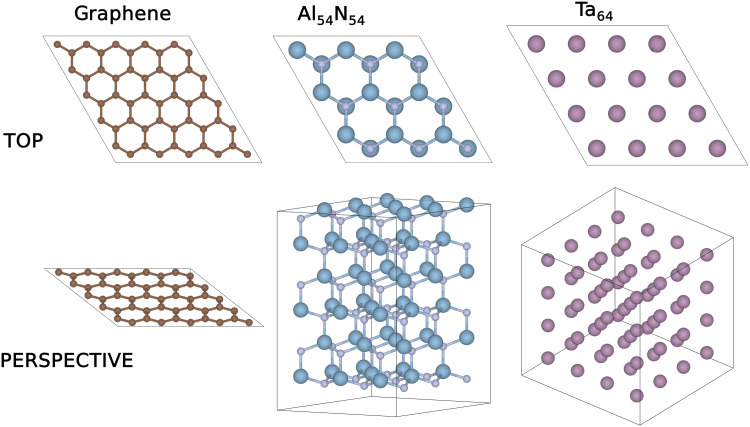
Graphene, AlN, and Ta supercells used in this work. The supercells contain 50, 108, and 64 atoms, respectively. For ${\text{Al}}_{1-x}{\text{Ga}}_{x}N$ , only the cation sublattice was required in the QUBO model. The cif files for these structures can be found in (*65*).

To show the versatility of the method presented in the main text, we have applied our approach to nitrogen-doped graphene (*28*, *29*), ${\text{Al}}_{1-x}{\text{Ga}}_{x}N$ (*30*, *31*), and ${\text{Ta}}_{1-x}W_{x}$ (*32*, *33*). The size of the supercells we used is limited by the available computer resources required to run the DFT training configurations, and here we used repeat cells containing 50 carbon atoms, 54 cations, and 64 metal sites, respectively, which are depicted in Fig. 6. With rapidly increasing computer resources, we anticipate that larger system sizes will be routinely explored using this efficient approach in the near future.

### DFT calculation details

The graphene and ${\text{Al}}_{1-x}{\text{Ga}}_{x}N$ DFT calculations were performed using the CRYSTAL23 code (*34*), which builds crystalline orbitals from a localized Gaussian basis function. The calculations used the B3LYP functional along with the revised pob-TZVP basis set (*55*), incorporating polarization, for all the elements. In the CRYSTAL23 code, the truncation criteria of the Coulomb and exchange infinite lattice series are controlled by five thresholds, which were set to 9 (T1 to T4) and 18 (T5). The SCF convergence threshold for the energy was set at 10^−8^ Hartree for the SCF convergence and 10^−6^ Hartree for structural relaxation. The reciprocal space sampling is based on a regular Pack-Monkhorst sublattice grid centered at the Γ point, with shrinking factors 12 and 24 for graphene calculations and 8 and 16 for ${\text{Al}}_{1-x}{\text{Ga}}_{x}N$ . The CRYSTALpytools (*56*) Python library was used to interact with the CRYSTAL23 input and output files and transform them into pymatgen objects for further postprocessing.

The VASP code was used for the TaW calculations. Using plane-wave basis functions, this software is better suited for the simulation of metals and metal alloys. We have used plane-wave DFT as implemented in VASP (*35*, *57*–*59*), using the Perdew-Burke-Ernzerhof (PBE) GGA functional (*60*), for electron exchange and correlation with the projector augmented wave method (*61*) to model the interaction between core and valence electrons (including five and six valence electrons for Ta and W, respectively). The total energy of the 64-atom fcc supercell was calculated using a 350-eV plane wave cut off and a 4 × 4 × 4 Γ-centered Monkhorst-Pack (*62*) *k*-point mesh, which provided convergence in total energy up to 2 × 10^−3^ eV (a 500-eV cutoff was used for relaxations of bulk structures). The elastic constants C_11_, C_12_, and C_44_ were computed using the finite displacement approach available in VASP, from which the bulk modulus was derived.

### The use of symmetry to train the QUBO model

Because DFT calculations can be computationally expensive, we aim to train the QUBO model from as few calculations as possible. To achieve this, we used the concept of SICs. In a supercell containing $N_{\text{sites}}$ fully occupied by $N_{\chi^{A}}$ and $N_{\chi^{B}}$ atoms, the number of possible configurations is

$$N_{\text{config}}=\frac{N_{\text{sites}}!}{N_{\chi^{A}}!N_{\chi^{B}}!}$$ (8)

depending on the symmetry of the material and size of the supercell, the $N_{\text{config}}$ configurations can be grouped into classes of symmetry equivalent configurations (SECs), where class *i* contains $N_{i}^{\text{SEC}}$ structures. By selecting one representative per class, we obtain the set of $N^{\text{SIC}}$ structures that represent all possible configurations within the supercell approximation. Several tools exist to calculate the SIC of a supercell, such as those reported by Grau-Crespo (*14*), D’Arco (*63*), and Mustapha *et al.* (*15*). The latter, implemented in CRYSTAL23 (*34*) via the CONFCOUNT keyword, is a linear-scaling method. However, the current implementation of CONFCOUNT is limited to supercells containing a maximum of 62 sites. Therefore, this approach was used in the nitrogen-doped graphene to obtain the full set of configurations up to a nitrogen atom concentration of 14%. The QUBO energy of these structures was used to calculate the Boltzmann distribution from an exhaustive search. This is compared with the probability distribution resulting from the quantum annealing run in different chemical potential temperature regimes reported in Fig. 4.

To train the QUBO model for ${\text{Al}}_{1-x}{\text{Ga}}_{x}N$ and ${\text{Ta}}_{1-x}W_{x}$ , we generate the structures randomly, ensuring that they are symmetry independent. Then, we select only a subset of SICs comprising $N_{\text{train}}^{\text{SIC}}$ structures. For each SIC, we calculate its DFT energy upon geometry relaxation at constant pressure and assign it to all the $N_{i}^{\text{SEC}}$ structures in the same symmetry class. The set of all SECs, containing $N_{\text{train}}^{\text{SEC}}$ structures, is used to train the QUBO model. The same procedure is repeated for the structures used to test the model.

Using this symmetry-enhanced approach, we obtain a relatively large training set from a very limited number of DFT calculations. The size of these subsets is reported in table S1. The $N_{\text{train}}$ and $N_{\text{test}}$ structures used to train and test the model cover the range of compositions of interest for the different materials ( $C_{49}N_{1}$ to $C_{40}N_{10}$ , ${\text{Al}}_{53}{\text{Ga}}_{1}N_{54}$ to ${\text{Al}}_{1}{\text{Ga}}_{53}N_{54}$ , and ${\text{Ta}}_{1}W_{63}$ to ${\text{Ta}}_{63}W_{1}$).

### Selecting the desired composition

The QUBO model is an unconstrained optimization technique. Constraints, such as the composition of structures, can be imposed through penalty functions. In our previous work [Section F in (*36*)], we demonstrated the use of penalty functions to enforce the desired number of vacancies in graphene. Imposing a constraint on the composition of the returned structures results in a fully connected QUBO model, which scales poorly on current QAs, as depicted in Fig. 2. On the other hand, the biases $Q_{\mathrm{ii}}$ are already present in the model and changing their values will not affect the mapping of the problem to the hardware. In this section, we discuss how, using the concept of chemical potential, we can tune the composition of the configurations returned by the QA by modifying the diagonal terms of the QUBO model only.

In the main text, we presented a simple way of obtaining Δμ in the case of a two-component material. Here, we discussed a more general derivation based on thermodynamic considerations. In general, the chemical potential of species $\chi^{A}$ in phase *i* is defined as

$$\mu_{\chi^{A}}^{i}={\left(\frac{\partial G}{\partial n_{\chi^{A}}^{i}}\right)}_{T,P}$$ (9)

where *G* is the Gibbs free energy, and $n_{\chi^{A}}^{i}$ is the number of particles (or moles) of species $\chi^{A}$ in phase *i*. The chemical potential represents the energy gain/loss when one particle (atom, formula unit, or mole) of species $\chi^{A}$ moves from one phase to another. The reservoir in our grand canonical ensemble contains both the $\chi^{A}$ and the $\chi^{B}$ species.

The partition function in the grand canonical ensemble is

$$\Xi =\sum_{i=0}^{N_{n}}M_{i}\text{exp}\frac{-\Delta {\tilde{E}}_{i}^{f}+\sum_{\chi_{j}}N_{\chi_{j}}\mu_{\chi_{j}}}{k_{B}T}=\sum_{i=0}^{N_{n}}M_{i}\text{exp}\frac{-\Delta E_{i}^{f}}{k_{B}T}$$ (10)

where $\Delta E_{i}^{f}$ and $M_{i}$ are the formation energy (Eq. 3) and the multiplicity of configuration *i*, and $N_{\chi_{j}}$ and $\mu_{\chi_{j}}$ are the number of atoms $\chi_{j}$ and its chemical potential in the reservoir. The probability of observing the system in state *i* is defined as

$$p_{i}=\frac{1}{\Xi }M_{i}\text{exp}\frac{-\Delta E_{i}^{f}}{k_{B}T}$$ (11)

The material and the reservoir form a closed system, indicating that the total number of atoms $\chi^{i}$ remains constant

$$N^{\chi^{i}}=N_{m}^{\chi^{i}}+N_{r}^{\chi^{i}}$$ (12)

where $N^{\chi^{i}}$ is the total number of $\chi^{i}$ atoms, $N_{m}^{\chi^{i}}$ is the number of $\chi^{i}$ atoms in the material, and $N_{r}^{\chi^{i}}$ is the number of $\chi^{i}$ atoms in the reservoir. In a binary material, each site in the structure is occupied by either an atom $\chi^{A}$ or $\chi^{B}$

$$N_{m}=N_{m}^{\chi^{A}}+N_{m}^{\chi^{B}}$$ (13)

where $N_{m}$ is the total number of sites in the material, and $N_{m}^{\chi^{A}}$ and $N_{m}^{\chi^{B}}$ are the number of $\chi^{A}$ and $\chi^{B}$ atoms in the material, respectively. The energy of a $\chi^{A}$ atom in the material is denoted by $\varepsilon^{\chi^{A}}$ , whereas the energy of a $\chi^{B}$ atom is represented by $\varepsilon^{\chi^{B}}$.

We define the total energy of the system (material and reservoir) as

$$E^{\text{TOT}}=\underset{\text{material}}{\underbrace{N_{m}^{\chi^{A}}\varepsilon^{\chi^{A}}+N_{m}^{\chi^{B}}\varepsilon^{\chi^{B}}}}+\underset{\text{reservoir}}{\underbrace{N_{r}^{\chi^{A}}\mu^{\chi^{A}}+N_{r}^{\chi^{B}}\mu^{\chi^{B}}}}$$ (14)

$$\begin{array}{c} E^{\text{TOT}}=\underset{\text{material}}{\underbrace{\left(N_{m}-N_{m}^{\chi^{B}}\right)\varepsilon^{\chi^{A}}+N_{m}^{\chi^{B}}\varepsilon^{\chi^{B}}}}+\underset{\text{reservoir}}{\underbrace{\left(N^{\chi^{A}}-N_{m}-N_{m}^{\chi^{B}}\right)\mu^{\chi^{A}}+N_{r}^{\chi^{B}}\mu^{\chi^{B}}}} \end{array}$$ (15)

Using Eq. 15, we can determine the change in energy with respect to the number of atoms $\chi^{B}$ in the material

$$\frac{\partial E^{\text{TOT}}}{\partial N_{m}^{\chi^{B}}}=\underset{\text{constant}>0}{\underbrace{-\varepsilon^{\chi^{A}}+\varepsilon^{\chi^{B}}}}\underset{\Delta \mu }{\underbrace{-\mu^{\chi^{A}}+\mu^{\chi^{B}}}}$$ (16)

This equation links the change in the concentration of $\chi^{B}$ atoms in the material with its chemical potential. The concentration of $\chi^{B}$ atoms predicted by the QA can therefore be controlled by adjusting the difference in the chemical potential, $\Delta \mu =\mu_{\chi^{B}}-\mu_{\chi^{A}}$.

**Fig. 7.**
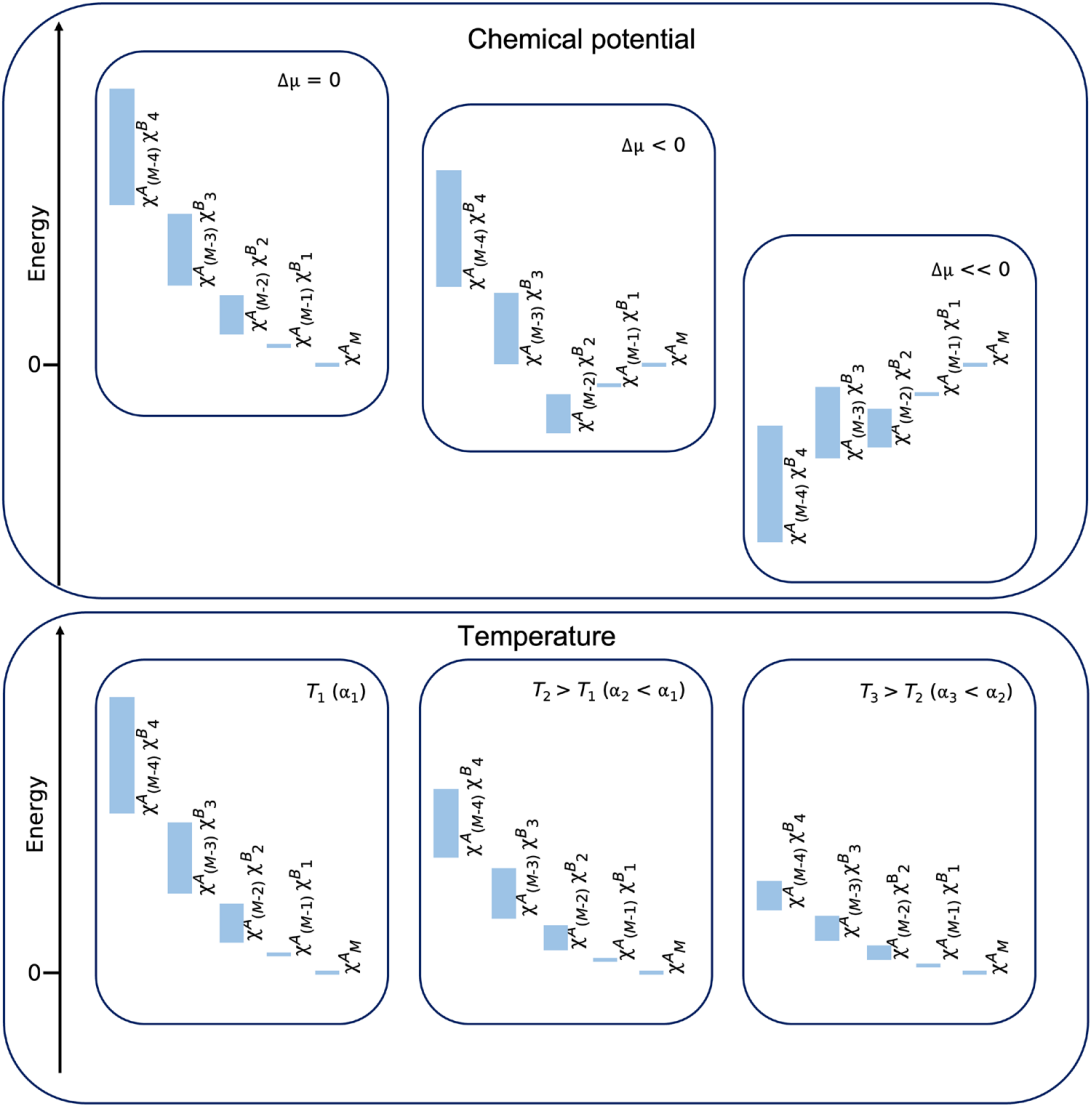
Schematic representation of the effect of the chemical potential ( Δμ ) and temperature on the QUBO energy levels. In the panel titled “Chemical potential,” Δμ decreases from left to right. With this decrease in chemical potential, materials that have higher concentrations of $\chi^{B}$ atoms become more energetically favorable. In the panel titled “Temperature,” the term α decreases from left to right, which corresponds to an increase in the temperature of the Boltzmann distribution we are simulating.

The effect of Δμ on the QUBO energy levels is depicted in the panel titled “Chemical potential” of Fig. 7. Going from left to right, we plot the schematic energy levels corresponding to larger negative values of Δμ . Using Δμ = 0, the lowest state energy is zero, which corresponds to the trivial solution of the structure containing only $\chi^{A}$ atoms. As the magnitude of the potential increases, materials that have higher concentrations of $\chi^{B}$ atoms become more energetically favorable.

### Introducing the temperature in the QUBO model

The effect of temperature on the probability distribution is introduced in the QUBO model through the scaling factor α, which can take values between 0 and 1. Before running on a QA, the D-Wave API Ocean transforms the QUBO model into an Ising Hamiltonian

$$\begin{array}{c} E\left(s\right)=\sum_{i}h_{i}s_{i}+\sum_{j>i}J_{\mathrm{ij}}s_{i}s_{j}\;s_{i}\in \left\{\;+1,-1\;\right\} \end{array}$$ (17)

by using $s_{i}=(2x_{i}-1)$ . In D-Wave annealers, the standard approach is to maximize $h_{i}$ and $J_{\mathrm{ij}}$ by using their entire possible value range. In the D-Wave Advantage QPUs, these are $h_{i}\in \left[-4,+4\right]$ and $J_{\mathrm{ij}}\in \left[-1,+1\right]$ . This procedure increases the energy difference between the ground state and the excited states. Therefore, it maximizes the probability of obtaining the ground state at the end of the anneal. This corresponds to the low temperature regime, where, according to the Boltzmann distribution, ground-energy states are predominant. If finding the ground state (the global minimum of the configurational energy landscape) is the goal of our simulation, this setting can be used. However, if we are interested in exploring probability-temperature distributions, we need to introduce a fictitious temperature in our Ising model. This can be achieved by deactivating the automatic scaling option (auto_scale = False) and scaling the values in the Ising model so that the effective range for $h_{i}$ and $J_{\mathrm{ij}}$ decreases. In this regime, the energy difference between the ground state and excited states can be tuned. When the parameter α is sufficiently small, nonadiabatic transitions are observed. In the main text, we discuss how the resulting distribution of configurations can be linked to a Boltzmann-like distribution.

The effect of the temperature on the QUBO energy levels is depicted in the “Temperature” panel of Fig. 7. Going from left to right, we plot the schematic energy levels corresponding to higher temperatures (smaller α). Unlike changes in the chemical potential, varying the temperature does not change the ranking of the configurations in terms of their energy. With an increase in temperature, we observe two effects on the energy levels. First, the energy difference between structures with varying concentrations of $\chi^{B}$ atoms becomes smaller. Second, the energy range for different configurations of species $\chi^{A}$ and $\chi^{B}$ within a fixed composition is also reduced.

### Building the QUBO model

In the QUBO model used in this study, the **Q** matrix (Eq. 1) incorporates all the interactions that define the energy of a structure as a function of the occupancy of its sites. In our previous work (*36*), which focused on the QA-assisted simulation of vacancies in graphene, we built the QUBO matrix based on the knowledge that a vacancy will result in broken bonds that will increase the energy of the structure. Here, we aim to build a QUBO matrix that can be used to calculate the energy of real-world materials. We achieve this by training the QUBO model using data obtained from DFT calculations and minimizing the following expression

$$\sum_{k}{\left(\Delta E_{k}^{f}-\sum_{i}\sum_{j\geq i}Q_{\mathrm{ij}}A_{\mathrm{ij}}^{\mathrm{kn}}\right)}^{2}$$ (18)

where *k* labels the configurations used in the training set and we use the adjacent matrix with elements $A_{\mathrm{ij}}^{\mathrm{kn}}$ defined as 1 if *i* and *j* are closer than (n+1)-th nearest neighbors and 0 otherwise. The formation energy $\Delta E_{k}^{f}$ is defined in Eq. 3.

We tested the effect of the number of neighbors, defined by the $A^{\mathrm{kn}}$ matrix, included in the model on the precision of the energy calculated using the QUBO model with respect to the DFT energy (see table S2 in the Supplementary Materials). Including more neighbors in the matrix $A^{\mathrm{kn}}$ results, in theory, in a more refined energy model because more interactions are included. However, the model also incurs overfitting. Furthermore, including more elements in the $A^{\mathrm{kn}}$ matrix results in a more connected QUBO model. For the results reported in the main text, we used $A^{\mathrm{kn}}$ (interactions up to the next-nearest neighbors) when training the QUBO model, which represents a compromise between the accuracy of the model and the number of couplings.

### Quantum annealing parameters

All quantum annealing calculations were performed using the D-Wave Advantage QPU through Leap using the associated Ocean Python API (*37*). The mapping of the QUBO (Ising) problem to hardware was performed using the minorminer library. We used the default values for the annealing time (20 μs) and the chain strength calculated using the uniform torque compensation method. For each of the results reported in this article, quantum annealing was run 1000 times. Therefore, each probability is calculated from a sample of 1000 configurations.
