# Supplementary material for: Exploring the thermodynamics of disordered materials with quantum computing
Source: Sci Adv. 2025 Jun 6;11(23):eadt7156. doi: 10.1126/sciadv.adt7156 (PMC12143349; doi:10.1126/sciadv.adt7156)
Supplement: Supplementary file 1 — Supplementary Text Tables S1 and S2 [file sciadv.adt7156_sm.pdf]

Science Advances
AAAS

Supplementary Materials for

Exploring the thermodynamics of disordered materials with quantum computing

Bruno Camino *et al.*

Corresponding author: Bruno Camino, b.camino@ucl.ac.uk; Scott M. Woodley, scott.woodley@ucl.ac.uk

Sci. Adv. **11**, eadt7156 (2025)
DOI: 10.1126/sciadv.adt7156

This PDF file includes:

Supplementary Text
Tables S1 and S2

Supplementary Text

Training the QUBO model

In Table S2, we report the mean absolute error (MAE) obtained for the three materials studied in this work using $\mathbf{A}^{k1}$-$\mathbf{A}^{k4}$. Including more elements in the $\mathbf{A}^{kn}$ matrix results, in theory, in a more refined energy model because more interactions are included. However, the model also incurs overfitting. This can be observed, for example, for graphene going from $\mathbf{A}^{k3}$-$\mathbf{A}^{k4}$.

Another factor to take into account, when selecting n, is the total number of couplings Q_{ij} within the QUBO matrix. As discussed above, an increased count of these couplings results in a model that does not scale efficiently on current quantum annealers. The two main sections of the supplement can be split up using headings.

	$N_{\text{train}}^{\text{SIC}}$	N_{train}	$N_{\text{test}}^{\text{SIC}}$	N_{test}
Graphene	10	2600	18	4550
$Al_{1-x}Ga_xN$	51	16092	48	6624
$Ta_xW_{(1-x)}$	61	178240	61	178240

Table S1: Size of the test and train sets. Only $N_{\text{train}}^{\text{SIC}}$ and $N_{\text{test}}^{\text{SIC}}$ DFT calculations are needed to train and test the QUBO model.

Graphene			
	R^2	MAE (eV/fu)	N. of couplings
$\mathbf{A^{k1}}$	0.9976	3.042×10^{-6}	75
$\mathbf{A^{k2}}$	0.9994	1.253×10^{-6}	225
$\mathbf{A^{k3}}$	0.9999	1.153×10^{-6}	300
$\mathbf{A^{k4}}$	0.9999	3.200×10^{-6}	450
$Al_{1-x}Ga_xN$			
	R^2	MAE (eV/fu)	N. of couplings
$\mathbf{A^{k1}}$	0.957	5.605×10^{-4}	75
$\mathbf{A^{k2}}$	0.957	3.919×10^{-4}	225
$\mathbf{A^{k3}}$	0.957	4.953×10^{-4}	300
$\mathbf{A^{k4}}$	0.957	3.185×10^{-4}	450
$Ta_{1-x}W_x$			
	R^2	MAE (eV/fu)	N. of couplings
$\mathbf{A^{k1}}$	0.9994	5.994×10^{-6}	256
$\mathbf{A^{k2}}$	0.9994	5.993×10^{-6}	448
$\mathbf{A^{k3}}$	0.9994	6.045×10^{-6}	832
$\mathbf{A^{k4}}$	0.9994	6.097×10^{-6}	1600

Table S2: The coefficient of determination R^2, mean absolute error (MAE) in units of eV/formula unit and the number of couplings in the QUBO model is reported for graphene, $Al_{1-x}Ga_xN$ and $Ta_{1-x}W_x$ when using adjacency matrices $\mathbf{A}^{k1}$ to $\mathbf{A}^{k4}$.
